# 

**Published:** 1891-08-08

**Authors:** 


PRICE ONE PENNY.

No. 254, Vol. X.—August 8th, 1891.

[Registered at the General Post Office as a Newspaper for transmission in the United Kingdom and Abroad.]

WITH EXTRA NURSING SUPPLEMENT.

Per Annum, 6s. 6d.; post free.

Monthly Parts, 6d.; post free 8d.

Ditto per Annum, 8s., post free.

### THE HOSPITAL

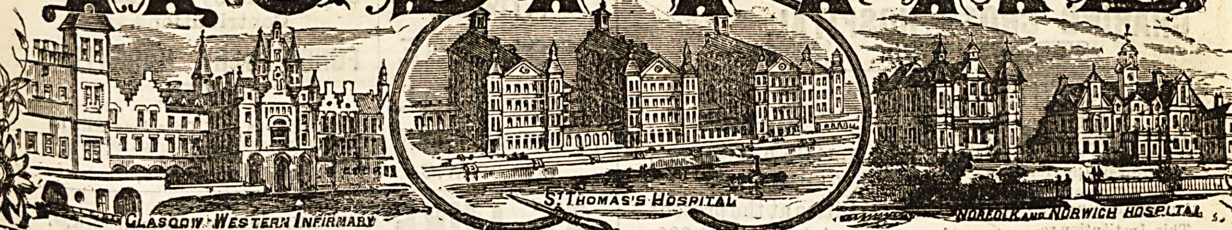

A WEEKLY JOURNAL OF

### Science, Medicine, Nursing and Philanthropy.

SATURDAY, AUGUST 8th, 1891.

| | |
|---|---|
| **A Quarter of a Century of Medicine** | 217 |
| **Passing Topics.**—The Nude at the Seaside | 218 |
| **The Doctor's Armchair.**—Doctors and Directors | 219 |
| **The Story of the Insane from Year to Year** | 220 |
| **Everybody's Page** | 221 |
| **Notes and News** | 222 |
| **General Charities** | 223 |
| **Scraps and Gleanings** | 223 |
| **Annotations.**—Beware of the Jam Pot—Nurse Training and Certification—Midwives' Registration | 224 |
| **Editor's Letter-box.**—A Bureau of Information | 227 |
| **The Practitioner's Mirror and Retrospect**— | |
| Medicine.—Sugar of Milk | 225 |
| Surgery.—Diseases of the Fingers: B—Paronychia | 226 |
| Practitioner's Bookshelf.—Bacteria and their Products | 226 |
| New Drugs, Appliances, and Things Medical.—Kops' Ale—Patent Stopper for Poisons—Alfred Carter's Furniture and Appliances—Soda-Water | 227 |
| **Co-operative Production and Profit-Sharing** | 228 |
| **The Student of Medicine.**—On German Students (*continued*)—Appointments—Vacancies—Scholarships and Prizes—Pass Lists | |

EXTRA SUPPLEMENT—THE NURSING MIRROR.

| | |
|---|---|
| **En Passant.**—Obstetrical Society—Sister Frances—The Royal Red Cross—Miniature Charts—The Princess of Wales and the Nurses—Nurses and the Benevolent Fund | cvii |
| **Lectures on Surgical Ward Work and Nursing.** By Alex. Miles, M.B.Edin., C.M., F.R.C.S.E. XXXII.—Bandages for the Trunk and Head | cviii |
| **Examination Questions** | cviii |
| **Christmas Competitions—Appointments** | cviii |
| **For Reading to the Sick**—God's Tapestry | cix |
| **Wants and Workers** | cix |
| **More Revelations about Registration** | cix |
| **The Princess of Wales' Fund for Mrs. Grimwood** | cx |
| **Presentation** | cx |
| **Everybody's Opinion.** Work in Vancouver—Thanks to All—A Protest—Registration—Lodgings in London | cxi |
| **Notes and Queries** | cxi |
| **Amusements and Relaxation** | cxi |
| **The R. B. N. A.**—Extraordinary Tactics | cxii |

